# Quantitative assessment of cerebellar ataxia, through automated limb functional tests

**DOI:** 10.1186/s12984-019-0490-3

**Published:** 2019-02-27

**Authors:** Ragil Krishna, Pubudu N. Pathirana, Malcolm Horne, Laura Power, David J. Szmulewicz

**Affiliations:** 1 School of Engineering, Deakin University, Waurn Ponds, 3216 Australia; 2 Florey Institute of Neuroscience and Mental Health, Parkville, 3052 Australia; 3 Balance Disorders and Ataxia Service, Royal Victorian Eye and Ear Hospital, St Andrews Place, East Melbourne, 3002 Australia; 4 Cerebellar Ataxia Clinic, Caufield Hospital, Alfred Health, Caufield, 3162 Australia

**Keywords:** Finger-to-nose (FNT), Diadochokinesia (DDK), Heel shin test (HST), Fast fourier transforms (FFT), Principal component analysis (PCA)

## Abstract

**Background:**

Cerebellar damage can often result in disabilities affecting the peripheral regions of the body. These include poor and inaccurate coordination, tremors and irregular movements that often manifest as disorders associated with balance, gait and speech. The severity assessment of Cerebellar ataxia (CA) is determined by expert opinion and is likely to be subjective in nature. This paper investigates automated versions of three commonly used tests: Finger to Nose test (FNT), test for upper limb Dysdiadochokinesia Test (DDK) and Heel to Shin Test (HST), in evaluating disability due to CA.

**Methods:**

Limb movements associated with these tests are measured using Inertial Measurement Units (IMU) to capture the disability. Kinematic parameters such as acceleration, velocity and angle are considered in both time and frequency domain in three orthogonal axes to obtain relevant disability related information. The collective dominance in the data distributions of the underlying features were observed though the Principal Component Analysis (PCA). The dominant features were combined to substantiate the correlation with the expert clinical assessments through Linear Discriminant Analysis. Here, the Pearson correlation is used to examine the relationship between the objective assessments and the expert clinical scores while the performance was also verified by means of cross validation.

**Results:**

The experimental results show that acceleration is a major feature in DDK and HST, whereas rotation is the main feature responsible for classification in FNT. Combining the features enhanced the correlations in each domain. The subject data was classified based on the severity information based on expert clinical scores.

**Conclusion:**

For the predominantly translational movement in the upper limb FNT, the rotation captures disability and for the DDK test with predominantly rotational movements, the linear acceleration captures the disability but cannot be extended to the lower limb HST. The orthogonal direction manifestation of ataxia attributed to sensory measurements was determined for each test.

**Trial registration:**

Human Research and Ethics Committee, Royal Victorian Eye and Ear Hospital, East Melbourne, Australia (HREC Reference Number: 11/994H/16).

**Electronic supplementary material:**

The online version of this article (10.1186/s12984-019-0490-3) contains supplementary material, which is available to authorized users.

## Background

The cerebellum integrates afferent inputs from the vestibular system, the ocular system and the proprioceptive system to control movements of the trunk, limbs, eyes, head and those producing speech. Dysfunction of the cerebellum, known as ataxia, is recognized by its characteristic disturbance of movement. Clinicians subjectively grade the patient’s performance of specify motor tasks to assess cerebellar defects in axial, speech or appendicular movements. In this study we focus on the assessment of appendicular function for which commonly used motor tasks include the Finger-to-Nose Test (FNT), test for upper limb Dysdiadochokinesia (DDK) and Heel-Shin Test (HST). These tasks are based on the seminal observations of Gordon Holmes and others, beginning over 100 years ago. They have in common:

- Movements that require action across limb joints
- Repetition and rhythm in a manner that requires some accuracy in stopping and starting
- Postural stability as a platform to execute these movements

Clinicians assess the performance by focusing on the accuracy and rhythmicity of the movements. Accuracy of movements is assessed by the extent to which there is undershoot or overshoot of the trajectory and target [1–3]. The term Dysmetria [4, 5] is often used to describe this form of increased inaccuracy and implies a disturbance of displacement [6]. While the impairment is attributed to an inability to judge distance or scale, increased and variable execution time is often taken as a measure of the impairment instead [7–9], suggesting that velocity and acceleration are also disturbed. These assessments are made on movements such as the FNT (for the upper limb) and the HST (for the lower limb). These movements consist of repetitive movements between two targets: the patients move the finger between their nose and the examiner’s finger in the former and their heel between the ankle and the knee of the other leg in the latter [10]. Because of the larger inertial mass, postural stability (difficulty in maintaining the heel on the knee of the other leg) is assessed as well as the accuracy of maintaining it on the shin while sliding the heel from the ankle to knee of the other leg [11].

Rhythmicity is tested by asking the patient to perform a series of the same movement repetitively. A common example, used in this study, is to ask the subject to tap the back of one hand with the fingers of the other hand and then rapidly turn the tapping hand over and tap with the back of the fingers. This alternating tapping is maintained for several iterations with the focus on the rhythm accuracy and stability of the tap. Any repetitive movement can serve this purpose and, for example, repeating syllables (such as “ta”) can serve this purpose and impaired performance is often described as Dysdiadochokinesia [12]: or “abnormal succeeding movement” in Greek. More generally, the focus in assessment is on the accuracy of stopping one cycle and starting the next (detected as variability in rhythm of the movement), the variability in striking the recipient hand (more variability is abnormal) and the speed of performance (slowness increases with cerebellar dysfunction). At the bedside, clinicians use pattern recognition rather than any objective measurement and as described above, it is unclear whether the kinematic parameter should be displacement, velocity, acceleration or some other measurement of accuracy and timing. The bedside assessment of patterns is formalized by clinical scales, eg Scale for the Assessment and Rating of Ataxia (SARA), which are explicit about how the test should be performed and how abnormalities should be scored but this serves to reinforce pattern recognition.

The complexity of disability manifestations in human movement necessitate the engagement of accurate measurements and robust feature extraction techniques. The inertial measurements constitute angular velocity and linear acceleration in orthogonal directions with respect to a sensor based frame of reference. Earlier studies [13] show the use and the effectiveness of accelerometer data processing in the evaluation of cerebellar dysfunction. Multiple sensors can be cumbersome and limit the usability and uptake as a performance assessment tool for the tests [14]. Complex and expensive rehabilitation tools typically requiring relatively large infrastructures and technical expertise for operation have been used for measuring patient abnormalities −particularly in clinical settings. Use of single or multiple inertial sensors (IMU) for feature extraction is inherently more user friendly and likely to be adapted for assessment in non-clinical settings [15]. This indeed can potentially be included in remote rehabilitation programs as well as providing personalized health care with regular assessments while patients are in their natural environments. The techniques highlighted in these studies have limited success due to less effective feature selection employed in data classification [11]. Clinicians use peripheral tests for identifying cerebellar ataxia and performs FNT for identifying tremor and imbalance in coordination, and test for upper limb DDK and HST for identifying movement irregularities. These clinical observations are inherently subjective and generally require verification by means of other clinical assessments. Severity assessment and quantification of the disability is necessary for progressive treatment plans leading to recovery and disability management [16].

**Fig. 1:**
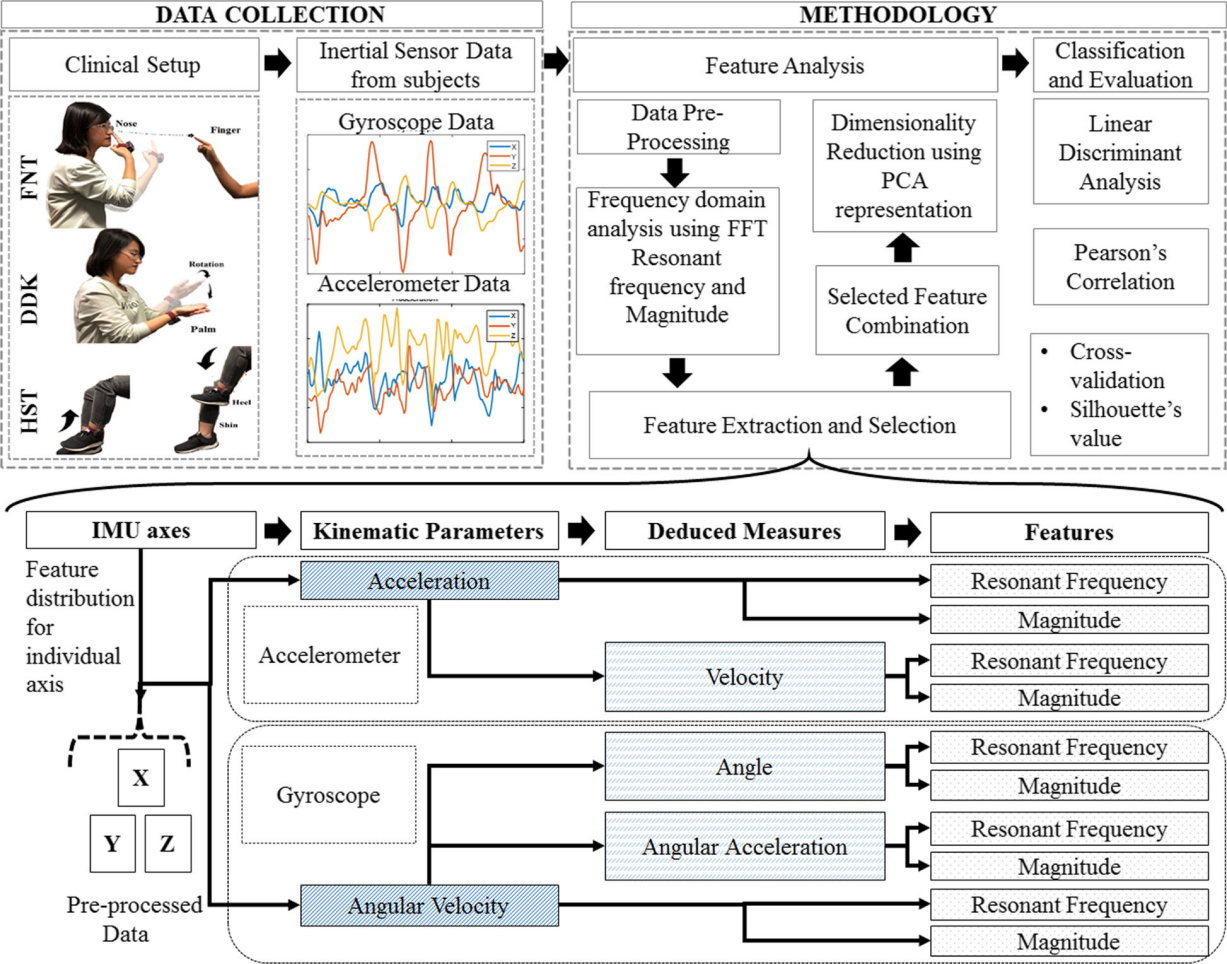
Schematic Representation of the Data Analysis: Feature Selection, Feature Extraction, Separation, Correlation and Classification

This work uses feature extraction techniques to identify features within the kinematic information gained from kinematic measures, that predict the presence and severity of ataxia as judged by the clinician. Clinician experienced in rating ataxia provided scores of the severity of ataxia as assessed by the performance of the test and also of the overall ataxia (all body parts).The *BioKin*^*TM*^ is 3 axes Inertial Measurement Units (IMU) with Wi-Fi communication for real time data capture and transmission. The sensory information captured from the tests is analyzed to uncover certain features intrinsically linked to the underlying disability as well as information describing the extent of the disability. The *BioKin*^*TM*^ sensors were attached securely to the relevant body part during the performance of the three tests. Linear acceleration and angular velocity were the kinematic measures captured from the sensor. The low pass filtered sensor kinematic information was subjected to frequency domain analysis using Fast Fourier Transforms (FFT). Data alignments observed through Principal Component Analysis (PCA) were initially used to reduce the dimensionality of the feature space and subsequently to enforce a balance between the clustering of the two dominant cohorts (patients and controls) and enhance the correlations with expert clinical scores through the Linear Discriminant Analysis (LDA) (Fig. 1). Further, machine learning approaches were used to enhance the classification.

## Methods

### Participants

There were 70 participants consisting of 31 people without ataxia (controls: 13 males and 18 females) aged 50 ± 25 years and, 39 people with ataxia of varying severity aged 60 ± 20 years. All subjects performed all three tests, and the movement data wais recorded using IMU sensors and accelerometry. All the participants signed informed consent forms and the study was approved by the Human Research and Ethics Committee, Royal Victorian Eye and Ear Hospital, East Melbourne, Australia (HREC Reference Number: 11/994H/16).

### Experimental Task

**Fig. 2:**
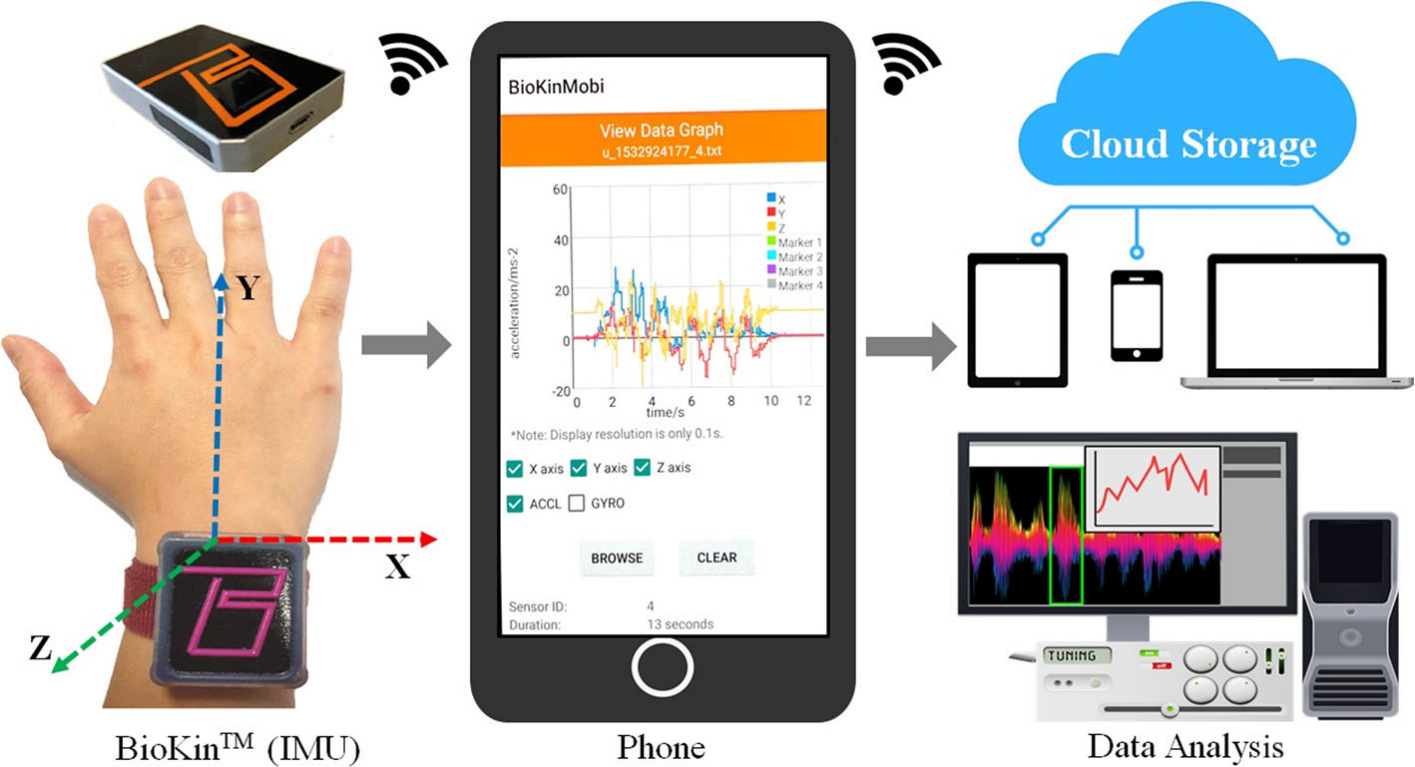
Data analysis using *BioKin*^*TM*^ sensor: The data is transmitted wirelessly to the phone and then to the cloud storage. This is available for the data analysts

Linear acceleration and angular velocity were captured using a *BioKin*^*TM*^ [17] wearable module that included a built-in MPU9250 IMU sensor and a IEEE802.11b/g/n/ wireless communication interface running on a 32-bit ARM processor while the FNT, upper limb test for DDK and HST were performed as given in Fig. 2.

**Fig. 3:**
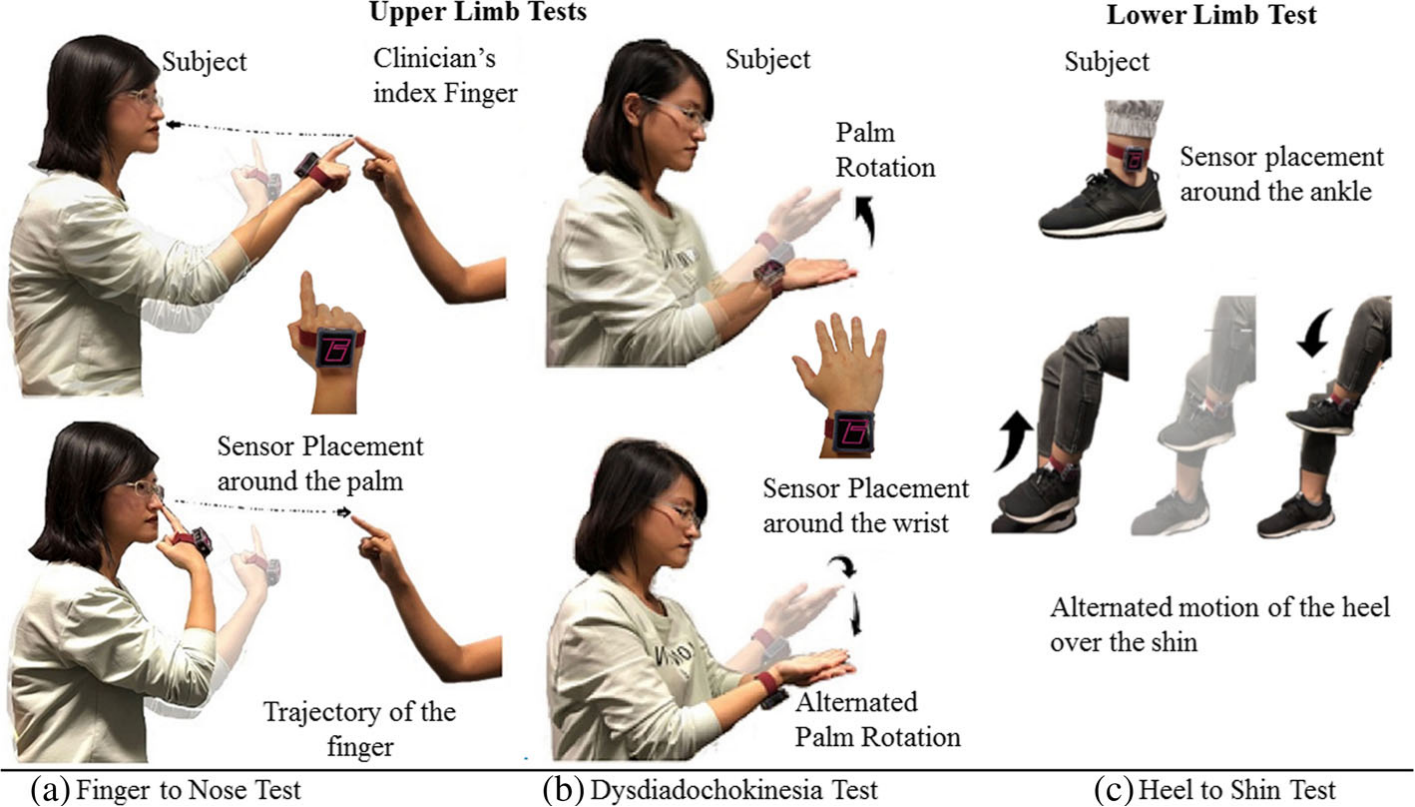
Tests for evaluation of cerebellar ataxia disorder: Finger to Nose, test for upper limb Dysdiadochokinesia, and Heel to Shin. The 3 tests were performed by all the participants

A. Finger to Nose Test: The subjects were instructed to touch the clinician’s index finger positioned in front of the patient. The subject was instructed to use their index finger to first touch the clinician’s finger and then their own nose and repeat this task for approximately 15 s (as depicted in Fig. 3a [18]. The test is performed for left and right limb consecutively. The clinician holds their finger at a stationary position during the task. The *BioKin*^*TM*^ unit is attached to the subject’s dorsal surface of the hand.

Test for upper limb Dysdiadochokinesia: The subject is instructed to place the dorsum of one hand on the palm of the other hand, as depicted in Fig. 3b. The subject is then instructed to pronate their hand, so that palm side faces downwards to rest on the palm of the other hand. The subject is also instructed to pronate and supinate between these two positions with maximum speed, and the rate of alternation is extracted from the *BioKin*^*TM*^’s IMU attached to the wrist [12].

B. Heel to Shin Test: With the subject in sitting position, they are instructed to place the heel of one foot onto the knee of the other leg (as indicated in Fig. 3c) and then slide the heel down the shin from the knee to the ankle and back up to the knee. This was repeated 10 times. The test is then transferred to the other leg. The clinician scores according to the accuracy and smoothness with which the heel maintains contact with the shin while performing the test. This motion is captured by the *BioKin*^*TM*^ tied around the ankle.

The clinical features of patients observed in this study, in comparison to three other studies [19] is given in Table 1.

**Table 1:** Clinical features of the patients

Clinical features	This study	Schöls	Ikeuchi	Matsumura
Number of patients	39	27	48	35
Age of onset	35+/- 20	30-71	35-67	28-73
Gender M/F	19/20			
Symptoms(%)				
Limb Ataxia	98	100	100	94
Gait Ataxia	60	100	96	100
Coordination Inaccuracy	53			
Spasiticity	53	35		3
Limb Dysmetria	48			92.8
Dysarthia	25	100	90	80
Positional Vertigo	25			

### Data Analysis

In cerebellar ataxia, translation and rotation can differ from controls in all three axis so each direction was analyzed separately. The gyroscope and accelerometer signals were sampled at 50Hz by the *BioKin*^*TM*^ sensor and we applied a further low pass filter with a cut-off frequency of 10 Hz to the *BioKin*^*TM*^ sensor output. A 6th order bandpass Butterworth Filter with a frequency band between 2Hz and 5Hz [20] gave better feature separation on inspection of the frequency bands 1-10Hz. This inspection selects the frequency band that gives maximum information.

With sensor bias removed, a 6th order bandpass Butterworth filter with frequency band between 5 Hz and 25 Hz is used for filtering noise frequencies to reduce the sensor drift.

The instantaneous sensor orientation (angle) of the wrist was calculated from the gyroscope measure [21]. The three main kinematic measures deduced were linear velocity from the accelerometer and, angular acceleration and angle of the body part from the gyroscope. Since the movements were designed to be alternating so as to capture a core feature of ataxia, measurement of their frequency was important. These specific kinematic features are extracted from the IMU measurements represents the disability effectively. Magnitude of acceleration exhibits different behaviours at different positions [22]. The mean, variance, root-mean square (RMS), and spectral energy coefficients of the signal magnitudes were parameters that formed part of the preliminary investigation as given in Table 2.

**Table 2:** Data Analysis using signal parameters

Parameters	FNT				DDK				HST			
Gyroscope												
	Controls		Patients		Controls		Patients		Control		Patients	
	Left	Right	Left	Right	Left	Right	Left	Right	Left	Right	Left	Right
Mean	226.85	204.7	149.8	161.6	384.5	381.89	279.86	283.1	287.33	287.2	185.8	181.1
Variance	1390.1	1951.	1915.	2207.	7000	5990	7590	5990	4500	4450	4580	4500
RMS value	229.89	209.4	156.1	168.3	393.5	389.6	293.1	293.53	290.55	289.2	210.1	210.0
Energy	860.4	856.3	1203.	1192.	908.4	907.7	1344.	1344.	880.4	877.7	1300.	1298.
Accelerometer												
Mean	10.97	10.78	10.39	10.43	11.95	11.96	11.24	11.33	11.34	11.33	11.01	10.97
Variance	0546	0319	0169	0331	2057	1633	1109	1542	1105	968	930	980
RMS value	11.00	10.79	10.40	10.45	12.0	12.03	11.29	11.40	11.54	11.22	11.17	11.124
Energy	-111.5	-110.	-139.	-130.	-114.	-113.8	-203.1	-203.5	-115.6	-114	-153.	-152.8

The filtered sensor readings were also analysed in the frequency domain using Fast Fourier Transforms (FFT). The resonant frequency (RF) and the magnitude of the frequency at resonance (MR) were captured as features from the peaks of the respective FFT waveform. Since there are 5 measures, the FFT analysis generated 60 features for the X, Y, Z considering both left and right limbs axes for each test and the task was to extract the features movement deficit for each test. Using hypothesis testing for *p*-values p < 0.05, the kinematic features of significance were selected. Correlation was considered reasonable for coefficient values ≥|0.5|. Accordingly, the dominant axes and the associated characteristic features were identified. Feature extraction techniques such as entropy and Dynamic Time Warping (DTW) were also engaged when evaluating the techniques outlined in this paper.

The features considered for each test were combined in conjunction with dimensionality reduction through the Principal Component Analysis (PCA), initially via the visual observation of data distributions along the principal axes components [23]. PCA technique essentially transforms the data into a new coordinate system by linear orthogonal transformation and projects the data as per the variance of distributions where the Diagonal covariance matrix maximizes feature variance. The principal components with maximum feature contribution were selected for further analysis. The PCA based feature selection conducted here was for the purpose of classification. Silhouette coefficient is used as a quantifiable measure of separation. It can be used to validate the data consistency within clusters. The average values show how well the groups are clustered in a tight space.

A detailed severity assessment of patients based on the extent of ataxia can intrinsically be linked to classification. A supervised classification technique using a multiclass Linear Discriminant Analysis (LDA) classifier was employed to evaluate the dominant data distribution derived using PCA to model the clinical scores of the severity of ataxia. The LDA data classification technique transformed the given data matrix into a lower dimension [24]. The data set comprising n-dimensional samples projected in a feature space was reduced onto a smaller subspace k for k <(n-1). The PCA generated feature space was used as input to LDA for the supervised learning approach. The LDA classifier discriminates the PCA features and categorized the subject data into three different classes- controls (severity score of “0”), patients with severity score of “1” with mild ataxia and patients with severity score of “2” with a significant level of ataxia, as a preliminary exercise to investigate the feasibility of uncovering clinically observed disability related information from intertial measurements. The classification of patients into the two severity classes was based on the assessments of three independent clinicians. In case of any discrepancies among clinicians, a majority decision was used, although no such instances were present for this patient cohort. There were 20 patients with score “1” and 19 patients with score “2”. The clinical validity of these scores was supported by the Scale for the Assessment and Rating of Ataxia (SARA) scores [25]. The classifier performance was assessed through the k-fold cross-validation technique. The underlying approach compared and evaluated algorithms while checking for over-fitting and potential misclassification issues. The linear discriminant model was developed using a dataset trained at *k*=10. The training data set was used to predict the testing data set labels. The accuracy, sensitivity and specificity of the discriminant model used for cross-validation was chosen as the performance evaluation criterion. These parameters represent the proportion of control subjects (specificity) and patients (sensitivity) that are correctly classified into the respective group.

## Results

Table 2 shows the temporal distribution of the inertial measurements by means of commonly used statistical parameters. These parameters suggest that rotational (gyroscope) kinematics are dominant for differentiating the two cohorts for the case of FNT. Spectral Energy is the common parameter in all 3 tests and demonstrate some differentiation between different cohorts- patient and control subjects consistently. The feature essentially corresponds to the energy content of the respective frequency bands in each test. Table 3 depicts the primary frequency domain features associated with the time series data for each test. The resonant frequency (RF), the magnitude of the resonant frequency (MR) and the kinematic parameters, for both left and right hand as well as for separate axes (X, Y and Z). The kinematic parameters include velocity (V) and acceleration (A) for the linear case, and angle (An), angular velocity (Av) and angular acceleration (Aa) for the rotational case. Together this amounted to a primary investigation of 60 features for each test.

**Table 3:** Pearson correlation values and *P*-values of extracted features

Test	D	L/R Pval	Angular Velocity		Acceleration		Angular Acceleration		Velocity		Angle	
			RF	MR	RF	MR	RF	MR	RF	MR	RF	MR
F N T												
	X	L	-0.2755	-0.1221	-0.2843	-0.2128	-0.0209	-0.4412	0.453	-0.0801	0.3231	0.0069
		Lp	0.61	0.54	0.72	0.65	0.58	0.75	0.64	0.75	0.62	0.67
		R	-0.3328	0.3288	-0.2325	0.192	-0.1777	-0.3841	0.5431	-0.237	0.1744	-0.3074
		Rp	0.64	0.55	0.63	0.59	0.51	0.71	0.61	0.72	0.58	0.54
	Y	L	-0.6599	-0.6763	-0.4151	-0.0256	0.3877	-0.6851	0.3183	-0.1454	0.568	-0.1897
		Lp	0.45	0.29	0.57	0.65	0.54	0.22	0.71	0.54	0.49	0.73
		R	-0.6582	-0.6777	-0.4269	-0.0738	0.3036	-0.6966	0.2043	-0.3542	0.589	-0.2105
		Rp	0.31	0.25	0.54	0.61	0.56	0.25	0.74	0.61	0.41	0.7
	Z	L	-0.2881	-0.2828	-0.3911	-0.1557	0.0485	0.3451	0.2723	-0.0567	0.3428	-0.0013
		Lp	0.52	0.51	0.58	0.71	0.52	0.66	0.59	0.61	0.78	0.73
		R	-0.4053	-0.2476	-0.295	-0.1278	0.2313	-0.3731	0.1654	-0.0294	0.3754	-0.3239
		Rp	0.56	0.53	0.6	0.78	0.56	0.53	0.68	0.58	0.78	0.74
D D K												
	X	L	-0.3294	-0.0339	0.5095	-0.5883	0.0411	-0.327	-0.3076	-0.3045	-0.0686	-0.1789
		Lp	0.54	0.71	0.43	0.29	0.72	0.52	0.59	0.68	0.58	0.72
		R	-0.4338	-0.0673	-0.5551	-0.5895	0.0316	-0.3031	-0.4736	-0.0984	0.344	-0.1012
		Rp	0.51	0.77	0.41	0.22	0.76	0.56	0.62	0.65	0.54	0.79
	Y	L	-0.6178	-0.5237	-0.2619	-0.0184	0.2253	-0.3416	-0.0388	-0.1986	-0.5635	-0.5846
		Lp	0.37	0.49	0.63	0.71	0.73	0.58	0.79	0.63	0.22	0.13
		R	-0.6202	-0.5072	-0.4719	-0.0387	0.2852	0.3865	-0.0461	-0.3757	-0.5532	-0.6143
		Rp	0.33	0.41	0.51	0.75	0.67	0.66	0.74	0.65	0.28	0.18
	Z	L	-0.3276	-0.1136	-0.6767	-0.5846	-0.0301	0.2416	-0.3045	-0.0846	0.1059	-0.3366
		Lp	0.52	0.67	0.11	0.35	0.76	0.55	0.56	0.72	0.74	0.58
		R	-0.1921	0.0378	-0.6958	-0.5511	-0.1492	-0.0865	-0.0606	-0.0511	-0.0511	0.0303
		Rp	0.56	0.69	0.25	0.32	0.74	0.72	0.79	0.81	0.7	0.53
H S T												
	X	L	-0.2458	-0.2792	-0.2426	0.2305	-0.1278	-0.3009	-0.3191	-0.0951	0.149	-0.1686
		Lp	0.6	0.71	0.65	0.67	0.65	0.52	0.59	0.75	0.67	0.78
		R	-0.2019	-0.2125	-0.1479	-0.0853	-0.1721	-0.2913	-0.2224	-0.2117	0.4766	-0.1781
		Rp	0.61	0.68	0.73	0.75	0.69	0.64	0.56	0.76	0.55	0.75
	Y	L	0.133	-0.3736	-0.2634	-0.5134	0.0058	-0.1071	0.3179	-0.3184	0.527	-0.131
		Lp	0.68	0.56	0.67	0.48	0.78	0.65	0.63	0.61	0.39	0.54
		R	-0.2129	-0.031	-0.1409	-0.5074	-0.0977	-0.3015	0.3121	-0.3732	0.5055	-0.1661
		Rp	0.66	0.54	0.66	0.49	0.81	0.61	0.56	0.64	0.29	0.53
	Z	L	-0.207	-0.2834	0.0865	-0.5677	0.1204	-0.2822	0.1363	-0.2127	0.397	-0.1521
		Lp	0.73	0.67	0.66	0.45	0.74	0.71	0.64	0.67	0.53	0.64
		R	-0.2725	-0.2394	-0.1536	-0.5912	-0.1643	-0.1214	0.0392	-0.2635	0.381	-0.1887
		Rp	0.65	0.59	0.83	0.41	0.64	0.66	0.67	0.62	0.64	0.68

D - Axes; Correlation: L-Left Limb, R-Right Limb; Lp- P value of Left Limb, Rp- P value of Right Limb; RF: Resonant Frequency MR: Magnitude of Resonant frequency

Using the hypothesis testing (*p*<0.05) denoted in Table 3, the features of significance indicated in Table 4 were identified for each test and for the purpose of clarity the following notational abbreviation for the features are adhered to: Frequency Feature $(RF or MR)_{(Test(L or R))}^{(Axis,Kinematic Parameter)}.$

**Table 4:** Feature of significance for each test

Test	Abbreviation	Description (L,R)
FNT	$RF_{FNT(L,R)}^{Y,A_{v}}$	RF of Av in Y-axis
	$MR_{FNT(L,R)}^{Y,A_{v}}$	MR of Av in Y-axis
	$RF_{FNT(L,R)}^{Y,A_{a}}$	RF of Aa in Y-axis
	$MR_{FNT(L,R)}^{Y,A_{a}}$	MR of Aa in Y-axis
	$RF_{FNT(L,R)}^{Y,A_{n}}$	RF of An in Y-axis
	$MR_{FNT(L,R)}^{Y,A_{n}}$	MR of An in Y-axis
DDK	$RF_{DDK(L,R)}^{Y,A_{v}}$	RF of Av in Y-axis
	$MR_{DDK(L,R)}^{Y,A_{v}}$	MR of Av in Y-axis
	$RF_{DDK(L,R)}^{X,A}$	RF of A in X-axis
	$MR_{DDK(L,R)}^{X,A}$	MR of A in X-axis
	$RF_{DDK(L,R)}^{Z,A}$	RF of A in Z-axis
	$MR_{DDK(L,R)}^{Z,A}$	MR of A in Z-axis
	$RF_{DDK(L,R)}^{Y,A_{n}}$	RF of An in Y-axis
	$MR_{DDK(L,R)}^{Y,A_{n}}$	MR of An in Y-axis
HST	$MR_{HST(L,R)}^{Y,A}$	MR of A in Y-axis
	$MR_{HST(L,R)}^{Y,A}$	MR of A in Z-axis
	$RF_{HST(L,R)}^{Y,A_{n}}$	RF of An in Y-axis
	$MR_{HST(L,R)}^{Y,A_{n}}$	MR of An in Y-axis

**Fig. 4:**
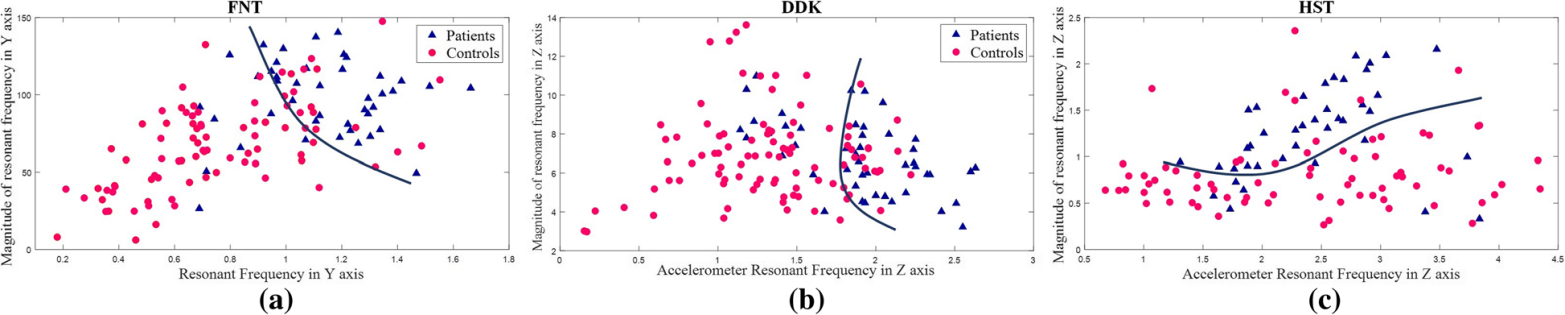
Resonant Frequency (RF) versus Magnitude (MR) from FFT analysis using features of high correlation. Figure 4**a** depicts Y-axis of gyroscope in FNT, Fig. 4**b** Z-axis of accelerometer in DDK, Fig. 4**c** depicts Z-axis of accelerometer in HST, and HST respectively

*Correlation:* The features with maximum correlation values given in Table 3 are plotted for each test to observe the reasonable seperation as depicted in Fig. 4. ${RF}_{(FNT(L,R))}^{(Y,A_{v})},{MR}_{(FNT(L,R))}^{(Y,A_{v})}$ are plotted in Fig. 4a for the FNT, with ${RF}_{(DDK(L,R))}^{(Z,A)},{MR}_{(DDK(L,R))}^{(Z,A)}$ in Fig. 4b and ${RF}_{(HST(L,R))}^{(Z,A)}$, ${MR}_{(HST(L,R))}^{(Z,A)}$ in Fig. 4c for DDK and HST respectively.

**Fig. 5:**
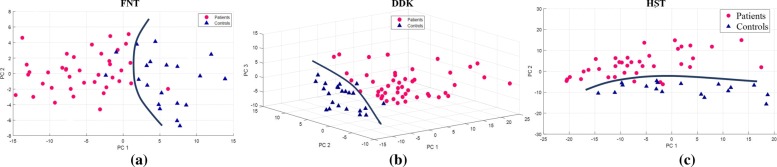
Best separation using PCA analysis of kinematic parameters. Figures 5**a**, **b**, **c** depicts the best PCA separation on feature combination for the FNT, DDK, HST respectively

*Separation:* The selected features in Table 4 were considered in terms of their ability to separate the two cohorts. Table 5 depicts the separation of the cohorts quantified in terms of Silhouette’s coefficient subjected to Principal Component Analysis (PCA). The ${RF}_{FNT(L,R)}^{(Y,A_{a})}$ and ${MR}_{FNT(L,R)}^{(Y,A_{a})}$ features in FNT combined generated the best separation as shown in Fig. 5a. Similarly,combining ${RF}_{DDK(L,R)}^{(X,A)},{MR}_{DDK(L,R)}^{(X,A)}$, ${RF}_{DDK(L,R)}^{(Z,A)}$ and ${MR}_{DDK(L,R)}^{(Z,A)}$ of acceleration data(X and Z axes) from the IMU in DDK generated good separation although the best separation is obtained when using the features ${RF}_{DDK(L,R)}^{(Z,A_{n})}$ and ${MR}_{DDK(L,R)}^{(Z,A_{n})}$ as shown in Fig. 5b.

**Table 5:** Measure of Separation using the Silhouette’s value (*S*_*v*_)

Parameters	FNT	DDK	HST
Acceleration	0.513	0.772*	0.793*
Angular Acceleration	0.784*	0.651	0.687
Velocity	0.535	0.421	0.725*
Angular Velocity	0.708	0.711	0.692
Angle	0.762*	0.783*	0.711*
All features combined	0.617	0.701	0.593
Upper Limb Combination	0.754	0.754	-
Preliminary Parameters (Accelerometer)			
Mean	0.305	0.282	0.319
Variance	0.222	0.274	0.334
RMS value	0.406	0.345	0.311
Energy	0.652	0.669	0.647
Preliminary Parameters (Gyroscope)			
Mean	0.528	0.277	0.294
Variance	0.505	0.246	0.299
RMS value	0.568	0.255	0.33
Energy	0.673	0.681	0.633
Other Domains (Max values):			
Entropy	0.533	0.487	0.479
Power Spectral Density	0.228	0.354	0.412
DTW	0.562	0.429	0.555

^*^indicates significant Silhouette values for separation

The ${MR}_{HST(L,R)}^{(Y,A)}$ and ${MR}_{HST(L,R)}^{(Z,A)}$ features combined gives separation in HST as depicted in Fig. 5c. The first 3 principal components PC1, PC2 and PC3 gave most of the information; 86%, 93% and 96% in 3 tests respectively. Separation is achieved in all tests using angle as the kinematic parameter, i.e, ${RF}_{FNT(L,R)}^{(Y,A_{n})}$ and ${MR}_{(FNT(L,R))}^{(Y,A_{n})}$ features gave separation in FNT, ${RF}_{(HST(L,R))}^{(Z,A_{n})}$ and ${MR}_{(HST(L,R))}^{(Z,A_{n})}$ gives separation in HST while ${RF}_{FNT(L,R)}^{(Y,A_{n})}$ and ${MR}_{(FNT(L,R))}^{(Y,A_{n})}$ was the best feature for DDK.

The Silhouette’s values (Sv) of the measures used in each test are given in Table 5 in accordance with the feature separation visualized using PCA.

DTW: Dynamic Time Warping The (*) marked values show the dominant separation, and the light highlighted values shows less prominent separation. Non-highlighted values represent weak or no separation.

**Fig. 6:**
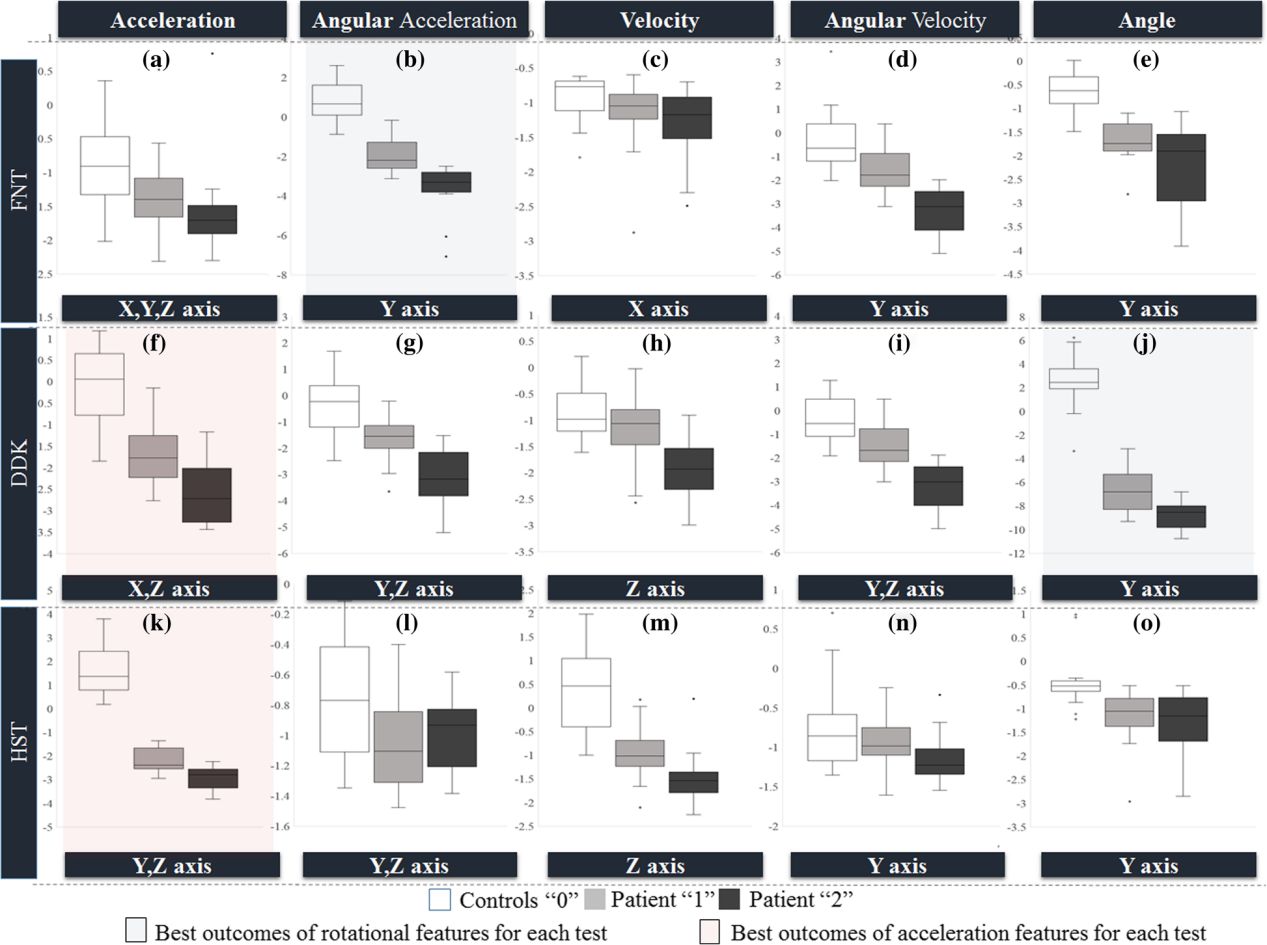
Boxplot representing the feature separation with doctors score using Linear Discriminant Analysis Classifier. The 3 different classes of the classifier include controls, patients with low severity and patients with high severity of ataxia. The panel labels indicate the axes showing best performance of the classifier for the five kinematic parameters in this study. In FNT (Fig. 6b), angular acceleration features along Y-axis gave superior classification compared to the other parameters. In DDK, X and Z-axis features of acceleration (Fig. 6f) and Y-axis features of angle (Fig. 6j) shows highest discrimination. In HST, acceleration features of Y,Z axis (Fig. 6k) discriminated the cohort of patients and healthy subjects compared to the other parameters. These best outcomes from the LDA analysis is highlighted separately for acceleration and rotation

*Classification:* All the selected features were considered for Linear Discriminant Analysis (LDA) for the purpose of classification into three different severity conditions. PCA combined features were used as an input for the classifier in each test as explained below:

Finger to Nose Test: The angular acceleration features ${RF}_{(FNT(L,R))}^{(Y,A_{a})}$ and ${MR}_{(FNT(L,R))}^{(Y,A_{a})}$ from the gyroscope using LDA as indicated in Fig. 6b gave the best classification of FNT. These features from both left and right limbs were combined using PCA to form the feature input to LDA. This classification provided the highest correlation with the clinical score (as given in Table 7: i.e., 0.7782). In Fig. 6a, linear acceleration as a feature generated only a marginal discrimination, when the resonant frequency and magnitude of X, Y and Z are combined as input features. The angular velocity, ${RF}_{(DDK(L,R))}^{(Y,A_{v})}$, ${MR}_{DDK(L,R)}^{(Y,A_{v})}$ as features gave noticeable classification as shown in Fig. 6d. This figure also infers that the angular velocity feature can primarily discriminate the groups with high severity patients (“2”) and no disability (“0’). In contrast the velocity feature (Fig. 6c) did not demonstrate acceptable correlation with the clinical score. ${RF}_{(FNT(L,R))}^{(Y,A_{n})}$ and ${MR}_{(FNT(L,R))}^{(Y,A_{n})}$ when using principal components as input to LDA in Fig. 6e resulted in noticeable classification relevant to severity.

Test for upper limb Dysdiadochokinesia: ${RF}_{DDK(L,R)}^{(X,A)}$, ${MR}_{DDK(L,R)}^{(X,A)}$, ${RF}_{DDK(L,R)}^{(Z,A)}$ and ${MR}_{DDK(L,R)}^{(Z,A)}$ features of the accelerometer resulted in superior discrimination and correlation with the clinician’s score as in Fig. 6f due to variations relevant to disability manifested in linear acceleration with regards to rate of rotation of the palm. These features indeed contribute to the maximum correlation value given in Table 7 for the test. The linear velocity features generated marginal discrimination in Fig. 6h and weak correlation with the clinical scores. ${RF}_{(DDK(L,R))}^{(Y,A_{v})}$ and ${MR}_{(DDK(L,R))}^{(Y,A_{v})}$ features showed reasonable separation in Fig. 6i while ${RF}_{(DDK(L,R))}^{(Z,A_{n})}$ and ${MR}_{(DDK(L,R))}^{(Z,A_{n})}$ features, showed convincing separation for DDK among the participants in terms of classification but lower correlation with clinical scores compared to the acceleration feature. The feature vector obtained from angle and acceleration can indeed discriminate the subject data independently while combining affected adversely.

Heel Shin Test:The ${MR}_{(HST(L,R))}^{(Y,A)}$ and ${MR}_{(HST(L,R))}^{(Z,A)}$ along Y, Z axes of the IMU resulted in the best separation for the HST using LDA classification as shown in Fig. 6k in addition to providing superior correlation as indicated in Table 7. The resonant frequency and magnitude of angular acceleration poorly discriminated the two cohorts in all axes (only Y, and Z is shown in Fig. 6l) while the magnitude of resonant frequency of velocity demonstrated reasonable separation (Fig. 6m). Further, angular velocity as a feature also failed to generate any visible separation as given in Fig. 6n. The ${RF}_{(HST(L,R))}^{(Y,A_{n})}$ and ${MR}_{(HST(L,R))}^{(Y,A_{n})}$ features captured the disability (separation of the two cohorts) while the severity manifestation was weak as indicated in Fig. 6o. Indeed, the acceleration feature generated the highest discrimination for the case of HST.

**Fig. 7:**
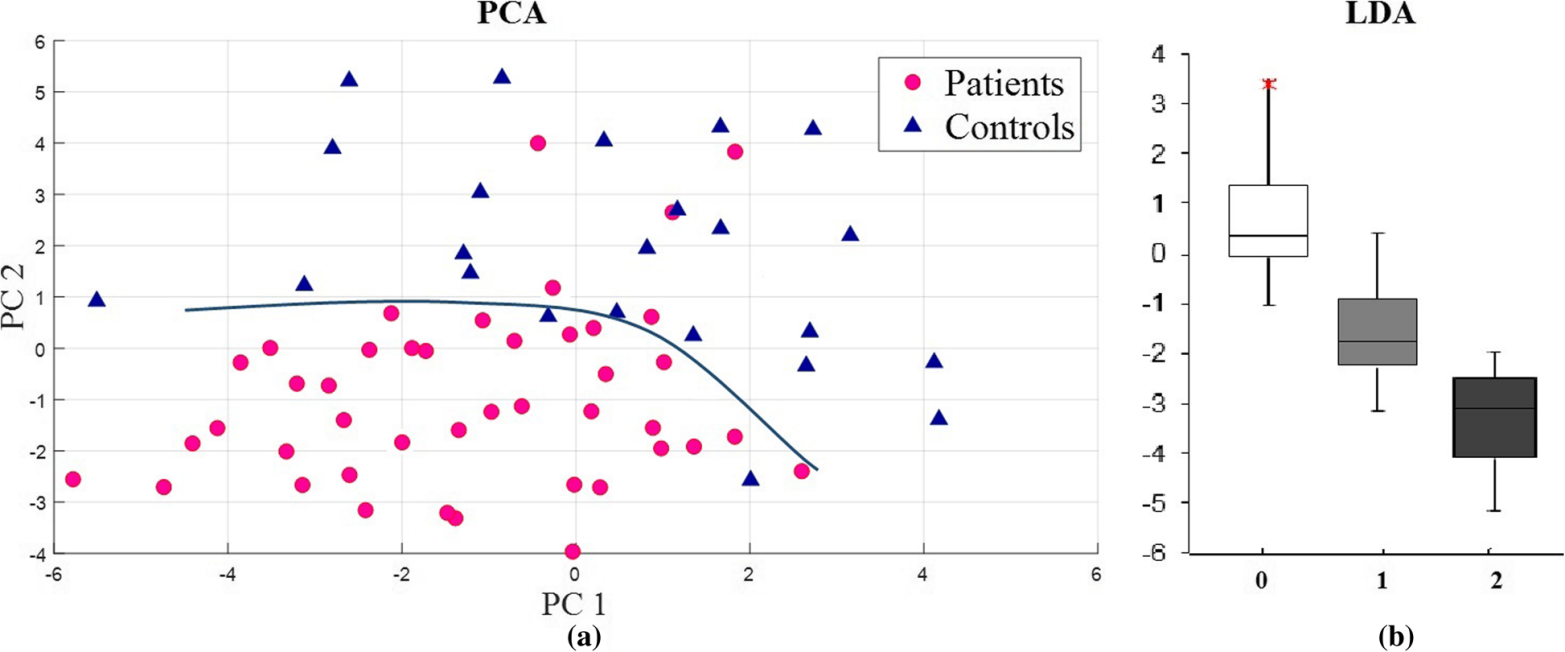
Combination of FNT and DDK test (upper limb). The upper limb tests on combination gave good separation as given in figure (**a**) and classified based on severity values as given in Figure (**b**)

Statistical parameters of the LDA outcomes (Fig. 6) are given in Table 6. The correlation of the LDA results with the clinical scores using Pearson correlation values are given in Table 7. The FNT and DDK (the two upper limb tests) were also combined using PCA resulting in a separation (Silhouette) value of 0.754 (Table 5) and classified using LDA resulting in an enhanced agreement with standard upper limb test correlation with an improved coefficient of 0.8253 (Table 7). The PCA separation and consequent LDA performance is shown in Fig. 7a and b respectively. Similarly, correlation of HST with lower limb tests scores gave a value of 0.7812 - no significant change from the correlation with the test specific score (0.7821 in Table 5) to the values given in Table 7. The clinician’s score is compared with standard SARA scores for each test with the use of Pearson correlation as given in Table 8.

**Table 6:** Statistical Analysis of the LDA outcomes

	FNT(Angular Acceleration) Fig. 6b			DDK(Angle) Fig. 6j			HST(Acceleration) Fig. 6k		
	Controls	Patients		Controls	Patients		Controls	Patients	
Severity	0	1	2	0	1	2	0	1	2
Minimum	-0.8306	-3.0897	-7.0334	-1.8815	-2.8054	-3.4728	0.2174	-2.908	-3.7858
Median	0.7073	-2.1527	-3.353	0.0239	-1.858	-2.8329	1.5675	-2.3297	-2.8275
Maximum	2.6476	-0.1296	-2.4498	0.9505	-0.1836	-1.2092	3.8271	-1.3204	-2.2008
Mean	0.8565	-1.943	-3.5043	-0.1537	-1.7186	-2.608	1.68132	-2.1615	-2.9156
Range	3.4782	2.9601	4.5836	2.832	2.6218	2.2636	3.6097	1.5882	1.5853
IQR	1.5167	1.0483	0.7305	1.2948	0.8618	1.2069	1.5673	0.7991	0.5468

IQR refers to the interquartile range and represents the spread/variance of the data

**Table 7:** Pearson Correlation Values (c) after applying LDA

	Frequency Domain	Entropy	DTW
Finger to Nose Test	0.7782	0.5668	0.5933
Diadochokinesia Test	0.8054	0.5192	0.6711
Heel to Shin Test	0.7821	0.5014	0.6512
Upper Limb Tests	0.8253	0.5122	0.4533
Lower Limb Test	0.7812	0.5080	0.4832

**Table 8:** Data and score (Clinician’s and SARA) correlations

TEST	Doctors Score 0-1-2	SARA score with Clinican’s Score	SARA correlation
FNT	0.7782	0.7542	0.7336
DDK	0.8054	0.7651	0.7254
HST	0.7821	0.7369	0.7055

**Fig. 8:**
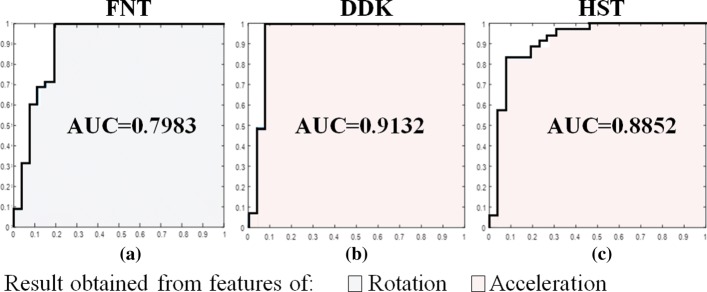
ROC curve for the 3 tests. The AUC values calculated from ROC curve is found to be 0.7983 for (**a**) FNT, 0.9132 for (**b**) DDK and 0.8852 for (**c**) HST

**Fig. 9:**
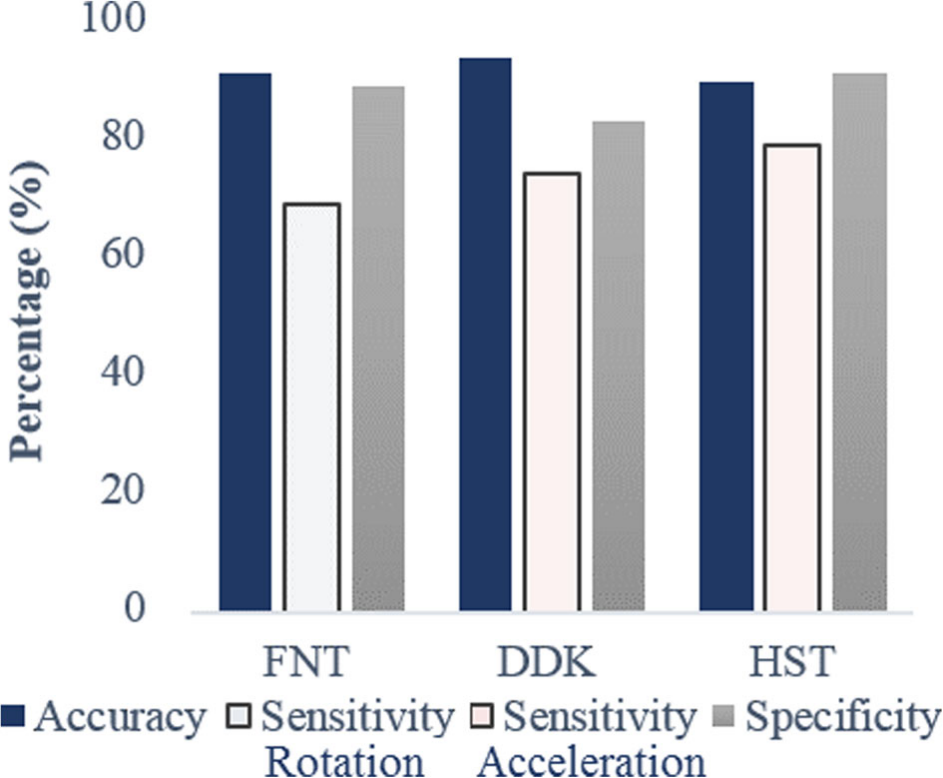
Cross Validation Parameters: Accuracy, Sensitivity and Specificity parameters for the 3 tests respectively

The outcome of the LDA classifier was cross validated to evaluate the classification performance. The Area under the Curve (AUC) values obtained from the Region Of Convergence (ROC) curve 0.7983, 0.9132 and 0.8852 for FNT, DDK and HST respectively are depicted in Fig. 8. True Positive Rate (number of patients accurately classified), False Positive rate accuracy (number of healthy subjects identified as patients) are stated in Table 9. These cross-validation parameters from Table 9 are presented in the form of accuracy, sensitivity and specificity in Fig. 9. The accuracy (max value = 1) values calculated from cross validation errors are, 0.9175, 0.9350 and 0.8955 for FNT, DDK and HST respectively. Specificity is given as 1-FPR, is above 80% for each of the tests. The selected features in terms of contribution for correlation is given in Table 10.

**Table 9:** True Positive Rate, False Positive rate, Error

	TPR	FPR	Error
FNT	0.69	0.11	0.2651
DDK	0.75	0.17	0.0683
HST	0.73	0.09	0.1045

**Table 10:** Features combination priority based on analysis

Test	Selected Features		PCA	LDA
FNT	${RF}_{FNT(L,R)}^{Y,A_{v}}$	C1	C2>C1>C3	C2>C1>C3
	${MR}_{FNT(L,R)}^{Y,A_{v}}$			
	${RF}_{FNT(L,R)}^{Y,A_{a}}$	C2		
	${MR}_{FNT(L,R)}^{Y,A_{a}}$			
	${RF}_{FNT(L,R)}^{Y,A_{n}}$	C3		
	${MR}_{FNT(L,R)}^{Y,A_{n}}$			
DDK	${RF}_{DDK(L,R)}^{Y,A_{v}}$	C4	C6>C5>C4	C5>C6>C4
	${MR}_{DDK(L,R)}^{Y,A_{v}}$			
	${RF}_{DDK(L,R)}^{X,A}$	C5		
	${MR}_{DDK(L,R)}^{X,A}$			
	${RF}_{DDK(L,R)}^{Z,A}$			
	${MR}_{DDK(L,R)}^{Z,A}$			
	${RF}_{DDK(L,R)}^{Y,A_{n}}$	C6		
	${MR}_{DDK(L,R)}^{Y,A_{n}}$			
HST	${MR}_{HST(L,R)}^{Y,A}$	C7	C7>C8	C7>C8
	${MR}_{HST(L,R)}^{Y,A}$			
	${RF}_{HST(L,R)}^{Y,A_{n}}$	C8		
	${MR}_{HST(L,R)}^{Y,A_{n}}$			

## Discussion

The feature selection using hypothesis testing (*p*-value) and the feature correlation using patient/control scores in Table 3 identified the kinematic parameters that best differentiated ataxia patients for each of the tests. Hence the characteristic features contributing to the disorder were obtained as a consequence of an exhausitive feature extraction process involving 60 features.

From Figs. 4, 5, 6 and Tables 5 and 6, the rotational motion provided better separation and correlation with FNT clinical scores. Similarly, for the case of DDK and HST acceleration-features distinguished control and patient data and correlated with clinical scores. From Fig. 4a, b and Table 3 resonant frequency as a (dominant) feature resulted in separation for the upper limb tests (FNT and DDK). The magnitude of the resonant frequency discriminated patients and controls in the lower limb test (HST) as indicated in Table 3, and Fig. 4c.

The rotational movement (via gyroscope) captures the disability resulting in greater separation for the case of FNT as shown in Tables 4 and 5. Patients repeated the tasks at a lower frequency than controls and this was evident from the frequency domain analysis as depicted in Fig. 4a. Angular acceleration along the Y-axis of the gyroscope (Fig. 6b) discriminated the two cohorts effectively. This can be associated with the uncoordinated movement of the arm while trying to reach the target. The rotation around Y-axis was during the repeated motion of the index finger from the nose to the target and then back to the nose. Thus, in FNT, gyroscope measure becomes the dominant criterion for discriminating the subject data based on severity.

In DDK test, the difference in the rotational angle between the two cohorts is captured through the resonant frequency and magnitude of angle variation. The range of the angle in the internal (pronation) and external rotation (supination) of the wrist differs for the two cohorts [26] resulting in movement bias intrinsic to the ataxia (Table 6). The rapidly alternating movements of one palm over the other resulted in movement abnormalities among the subjects along the Z-axis of the accelerometer as shown in Fig. 4b. Also, the patients executed the test at a relatively slower pace than the controls. This infers that the test for upper limb DDK differentiates ataxia patients based on the frequency of operation, linked to the difficulty in performing the alternating action as well as the range of rotation is offset to the controls in the angle variation [27]. The LDA results (Fig. 6f) obtained for the DDK test also establishes the inability of the patients to perform rapid alternating movements manifesting as linear acceleration in X, Z-axis. The patients are identified to have lower value of angular velocity along Y-axis and this represents their difficulty in rotating the palm while performing the test.

In Heel-to-Shin test, since the test mainly involves performing linear motion along a straight line in an alternating manner (up and down), the accelerometer components typically distinguished the subject data as shown in Fig. 4c. The acceleration features in Table 5 generated separation between control and patient cohorts. This can be related to the variation in the sliding motion of the heel while maintaining the contact with the shin. Also, the patient cohort performed the test at a lower magnitude of resonant frequency compared to the control cohorts. From Fig. 6k, acceleration features along X and Z axis produced the best separation and correlation with clinical score (Table 7) and the difference in direction of ataxia manifestation can be associated to the uneven motion of the leg along shin. Rotation along the Y-axis of the gyroscope is observed in all the three tests and separated controls and patients (not based on severity) by using resonant frequency and magnitude of the angle.

From Table 5, the Silhouette values suggest that the analysis employed in this paper generated better separation compared to the other existing techniques Sv = 0.793 (highest value). From Table 2, the preliminary analysis showed frequency domain analysis (spectral energy) gives dominant data separation and equivalent results are found from Tables 5 and 6. This implies that frequency domain analysis gave better results compared to the other feature extraction techniques.

From Tables 5, 6 and Fig. 7, the combination of the upper limb tests (FNT and DDK) improved the correlation with the clinician’s score. The separation value is Sv = 0.754 which falls under high separation coefficient and correlation of 0.8219 which is greater than individual correlation values for the tests. HST also correlated with the lower limb test scores value of 0.7812 comparable to the correlation with the HST test scores. The data correlates with both the clinical and SARA scores equally, as denoted in Table 8.

From Fig. 9, the performance parameters from cross-validation demonstrated a higher degree of classification into severity scores indicating a significantly higher correlation substantiated by the higher AUC and accuracy values. Hence, this analysis adopted using PCA and LDA supervised classification provided acceptable level of performance. DDK test and HST are observed to have superior values of classification performance parameters in comparison to that of FNT as depicted in Table 9 and Fig. 8. The results acquired in Table 9 have a higher degree of specificity which confirms greater success rate in using the model during cross-validation. The error rates of classification are found to be very low providing successful classification. Support Vector Machine is another supervised machine learning technique used for the analysis but since the number of subject data is limited (70), an extensive data analysis involving other learning-based approaches are planned for future analyses.

## Conclusion

An automated peripheral test for the objective assessment of Cerebellar Ataxia is investigated using an IMU based motion capture system during standard peripheral bedside tests.

The acceleration (linear/rotational) from the *BioKin*^*TM*^ was identified as the most prominent feature capturing the disability. In the case of FNT, the most convincing separation between patients and controls resulted by the combination of gyroscope features. In particular, angular acceleration is the dominant feature obtained with the best correlation with the clinical score. In contrast, for the DDK and HST tests, linear acceleration features resulted in the best separation and correlation with the clinical scores. It is notable that for the upper limb FNT, predominantly translational movement, the rotation captures disability and for the DDK test with predominantly rotational movements, the linear acceleration captures the disability although this cannot be extended to the lower limb HST. Contrastingly, HST does not consist of considerable limb rotation.

The evaluation of the three tests suggests that cerebellar ataxia is not manifested in the direction of the dominant limb motion during the course of the tests as shown in Table 11. The characteristic features that clinicians do not observe in normal bedside testing include features such as angle and the movements generated as artefacts from the primary motion of the test. Rotational movement around the Y-axis (pronation and supination) in the form of angle variation is dominant for all tests separating controls and patients. The FNT is considered a goal-oriented test while the DDK and HST are considered alternating movement tests.

**Table 11:** Manifestation of cerebellar ataxia through sensory means

Test	Kinematic parameter	Direction of movement deficit
FNT	Angular acceleration	Y
	Angle	Y
	Acceleration	X,Z
DDK	Angular velocity	Y
	Angle	Y
HST	Acceleration	Y,Z
	Angle	Y

In the alternative movement tests (DDK and HST), linear acceleration is observed as the dominant feature while for the goal-oriented test, rotational acceleration is observed as dominant. It is also notable that the goal oriented FNT is executed with the participation of one limb while for the alternating movements tests, both limbs participate for the test, although only one limb is primarily engaged in the execution of the test.

Frequency domain features generated better separation (Sv = 0.793) and correlation (c = 0.8219) for the tests compared to features in the time domain (DTW c = 0.6711, Sv = 0.562) and the entropy domain (c = 0.5668, Sv = 0.533). The validity of the clinical scores are further coincidentally supported, as the “1” and “2” SARA scores directly correlate with the clinician’s score of “1”. In the same respect, the SARA scores of “3” and “4” correspond to the clinician’s score of “2” for this patient cohort.

The combination of FNT and DDK gave better correlation with the clinical scores than independently. Therefore, the combination provided better correlation of the upper limb score. The HST correlated with clinical test scores (c = 0.7829) as well as lower limb scores (c = 0.7812). The combination of all three tests do not provide a full picture of ataxia (correlating with a global/generic score) as other domains are not covered under these peripheral tests.

## Additional files

Additional file 1 Data Analysis using signal parameters in page 5, Section: Data Analysis. (XLSX 10 kb)

Additional file 2 Pearson correlation values and *P*-values of extracted features in page 5, Section: Data Analysis. (XLSX 13.5 kb)

Additional file 3 Statistical Analysis of the LDA outcomes in page 9, Section: Results. (XLSX 10.3 kb)

## References

[1] Johansson GM, Grip H, Levin MF, Häger CK (2017). The added value of kinematic evaluation of the timed finger-to-nose test in persons post-stroke. J Neuroengineering Rehabil.

[2] Horak FB, Dimitrova D, Nutt JG (2005). Direction-specific postural instability in subjects with parkinson’s disease. Exp Neurol.

[3] Rodrigues MR, Slimovitch M, Chilingaryan G, Levin MF (2017). Does the finger-to-nose test measure upper limb coordination in chronic stroke?. J Neuroengineering Rehabil.

[4] Hore J, Wild B, Diener H (1991). Cerebellar dysmetria at the elbow, wrist, and fingers. J Neurophys.

[5] Schmahmann JD (2004). Disorders of the cerebellum: ataxia, dysmetria of thought, and the cerebellar cognitive affective syndrome. J Neuropsychiatry Clin Neurosci.

[6] Feys PG, Davies-Smith A, Jones R, Romberg A, Ruutiainen J, Helsen WF, Ketelaer P (2003). Intention tremor rated according to different finger-to-nose test protocols: a survey. Arch Phys Med Rehabil.

[7] Bastian A, Martin T, Keating J, Thach W (1996). Cerebellar ataxia: abnormal control of interaction torques across multiple joints. J Neurophys.

[8] Gagnon C, Mathieu J, Desrosiers J (2004). Standardized finger-nose test validity for coordination assessment in an ataxic disorder. Can J Neurol Sci.

[9] Kornegay J (1991). Ataxia, dysmetria, tremor. cerebellar diseases. Probl Vet Med.

[10] Obdrzálek S, Kurillo G, Ofli F, Bajcsy R, Seto E, Jimison H, et al.Accuracy and robustness of Kinect pose estimation in the context of coaching of elderly population. In: Conf Proc IEEE Eng Med Biol Soc. San Diego: 2012. p. 1188–1193. 10.1109/EMBC.2012.6346149 23366110

[11] Giggins OM, Sweeney KT, Caulfield B (2014). Rehabilitation exercise assessment using inertial sensors: a cross-sectional analytical study. J Neuroengineering Rehabil.

[12] Katz DI, Kreutzer JS, DeLuca J, Caplan B (2011). Dysdiadochokinesia. Encyclopedia of Clinical Neuropsychology.

[13] Taylor PE, Almeida GJ, Kanade T, Hodgins JK (2010). Classifying Human Motion Quality for Knee Osteoarthritis using accelerometers. 2010 Annual International Conference of the IEEE.

[14] Taylor PE, Almeida GJ, Hodgins JK, Kanade T. Multi-label classification for the analysis of human motion quality. In: Conference proceedings: Annual International Conference of the IEEE Engineering in Medicine and Biology Society. IEEE Engineering in Medicine and Biology Society. Annual Conference. Vol. 2012: 2012. p. 2214. 10.1109/EMBC.2012.6346402 23366363

[15] Giggins OM, Sweeney KT, Caulfield B (2014). Rehabilitation exercise assessment using inertial sensors: a cross-sectional analytical study. J Neuroeng Rehabil.

[16] Glass GV (1976). Primary, secondary, and meta-analysis of research. Educ Res.

[17] Ekanayake SW, Morris AJ, Forrester M, Pathirana PN (2015). Biokin: an ambulatory platform for gait kinematic and feature assessment. Healthc Technol Lett.

[18] Iverson GL, Kreutzer JS, DeLuca J, Caplan B (2011). Finger to nose test. Encyclopedia of Clinical Neuropsychology.

[19] Takahashi H, Ishikawa K, Tsutsumi T, Fujigasaki H, Kawata A, Okiyama R, Fujita T, Yoshizawa K, Yamaguchi S, Tomiyasu H (2004). A clinical and genetic study in a large cohort of patients with spinocerebellar ataxia type 6. J Hum Genet.

[20] Díez LE, Bahillo A, Masegosa AD, Perallos A, Azpilicueta L, Falcone F, Astrain JJ, Villadangos J. Signal processing requirements for step detection using wrist-worn IMU. In: Electromagnetics in Advanced Applications (ICEAA), 2015 International Conference on 2015 Sep 7. IEEE: 2015. p. 1032–1035.

[21] Fang B, Sun F, Liu H, Guo D. Development of a Wearable Device for Motion Capturing Based on Magnetic and Inertial Measurement Units J Scientific Programming, vol. 2017, Article ID 75947632. 2017; 2017:11.

[22] Incel OD (2015). Analysis of movement, orientation and rotation-based sensing for phone placement recognition. Sensors.

[23] Abdi H, Williams LJ (2010). Principal component analysis. Wiley Interdiscip Rev Comput Stat.

[24] Hochkirchen T (2010). Modern multivariate statistical techniques: regression, classification, and manifold learning. J R Stat Soc Ser A Stat Soc.

[25] Schmitz-Hübsch T, du Montcel ST, Baliko L, Berciano J, Boesch S, Depondt C, Giunti P, Globas C, Infante J, Kang J-S, Kremer B, Mariotti C, Melegh B, Pandolfo M, Rakowicz M, Ribai P, Rola R, Schöls L, Szymanski S, van de Warrenburg BP, Dürr A, Klockgether T. Scale for the assessment and rating of ataxia. 2006; 66(11):1717–20. 10.1212/01.wnl.0000219042.60538.92. 10.1212/01.wnl.0000219042.60538.92 16769946

[26] López-Nava IH, Márquez-Aquino F, Munoz-Meléndez A, Carrillo-López D, Vargas-Martínez H. Automatic measurement of pronation/supination, flexion/extension and abduction/adduction motion of human limbs using wearable inertial and magnetic sensors. In: Proc. 4th Int. Conf. Global Health Challenges (IARIA): 2015. p. 55–60.

[27] Callahan CD, Barisa MT (2004). Traumatic brain injury: Methods for clinical and forensic neuropsychiatric assessment. J Head Trauma Rehabil.

